# Complete mitochondrial genome of Gongshan muntjac (*Muntiacus gongshanensis*), a Critically Endangered deer species

**DOI:** 10.1080/23802359.2019.1660242

**Published:** 2019-09-06

**Authors:** Yun-Chun Zhang, Chen Xiao-Yong, Guo-Gang Li, Quan Rui-Chang

**Affiliations:** a Southeast Asia Biodiversity Research Institute, Chinese Academy of Sciences, Yezin, Nay Pyi Taw, Myanmar and Center for Integrative Garden, Chinese Academy of Sciences, Mengla, Yunnan, China;; b University of Chinese Academy of Sciences, Beijing, China;; c State key laboratory of Genetic Resources and Evolution, Kunming Institute of Zoology, Chinese Academic of Sciences, Kunming, Yunnan, China

**Keywords:** *Muntiacus gongshanensis*, mitochondrial genome, phylogenetics

## Abstract

The first complete mitochondrial genome of the Gongshan muntjac (*Muntiacus gongshanensi*s) was determined and annotated (GenBank accession nos. MK882935). The 16,356 bp circular genome contained 13 protein-coding genes (PGCs), 22 transfer RNA (tRNA) genes, 2 ribosomal RNA (rRNA) genes, and 1 control region (D-loop). Phylogenetic analysis revealed that Gongshan muntjac is most closely related to Black muntjac (*Muntiacus crinifrons*), with Fea’s muntjac (*Muntiacus feae*) as their sister species. These data will be useful for further studies on the genetic diversity and molecular phylogenetic relationship of the genus *Muntiacus*.

Gongshan muntjac (*Muntiacus gongshanensis*) is a medium-sized deer species, belonging to *Muntiacus*, Muntiacinae, Cervidae. This species was described from Gongshan County, Yunnan province, southwestern China (Ma et al. 1990). Gongshan muntjac is evaluated as Data Deficient (DD) in IUCN red list of Endangered species (Timmins and Duckworth 2016) and as Critically Endangered (CR) in the latest red list of China’s vertebrates (Jiang et al. 2016). However, molecular studies about Gongshan muntjac were limited and its complete mitochondrial genome has not been determined and characterized yet. The genetic relationship between Gongshan muntjac and other species is still not clear. Therefore, we report here the complete mitogenome of *M. gongshanensi*s and to clarify its relationships with other species of the genus *Muntiacus*.

The total genomic DNA was extracted (TIA Namp Genomic DNA Kit) from the muscle of Gongshan muntjac, collected from Gongshan County, Yunnan province, China. The sample and DNA (Accession number 1708001) were deposited in Center for Integrative Conservation, Xishuangbanna Tropical Botanical Garden, Chinese Academy of Sciences, Mengla, Yunnan, China. We designed 14 primers of the complete mitochondrial genome, referring to previous studies (Zhang et al. 2004; Li et al. 2017; Martins et al. 2017). All the primers synthesis and sequencing were done by Beijing Tianyi Huiyuan Bioscience and Technology Incorporation (Beijing, China). We aligned the sequences by default parameters in AliView (Larsson 2014), and mitochondrial genome was assembled using Geneious (Kearse et al. 2012). We used CIPRES implementation of maximum likelihood (ML) to analyze the phylogenetic relationship among muntjacs (https://www.phylo.org/portal2). Bayesian inference (BI) and phylogenetic tree construction were implemented in MRBAYES 3.2.1 software (Ronquist et al. 2012).

The complete mitochondrial genome of Gongshan muntjac is a circular with a length of 16,356 bp and was deposited in GenBank under accession nos. MK882935. The mitochondrial genes of Gongshan muntjac include 13 protein-coding genes, 2 rRNA genes, 22 tRNA genes, and 1 non-coding control region, most of which are encoded on a heavy strand, except for COX1, Cyt *b*, D-loop and 6 tRNAs (tRNA^Gln^, tRNA^Asn^, tRNA^Cys^, tRNA^Tyr^, tRNA^Asp^, and tRNA^Glu^). The overall base composition of the heavy strand is 33.2% A, 28.9% T, 24.5% C, and 13.4% G with a strong AT bias of 62.1%. The 22 tRNA genes are interspersed along the genome, with lengths varying from 60 to 75 bp and the inferred secondary structures of tRNA conform to the characteristic structural features of mitochondrial tRNAs. The lengths of 12S rRNA and 16S rRNA are 956 bp and 1569 bp, respectively. The D-loop region is located between tRNA^Pro^ and tRNA^Phe^ and is 923 bp in length. Phylogenetic analyses of 8 muntjac species based on two methods (ML and BI) displayed the consistent topology (Figure 1). We determine the Gongshan muntjac to be a member of genus *Muntiacus,* it is most closely related to Black muntjac, with Fea’s muntjac as their sister species.

**Figure 1.**
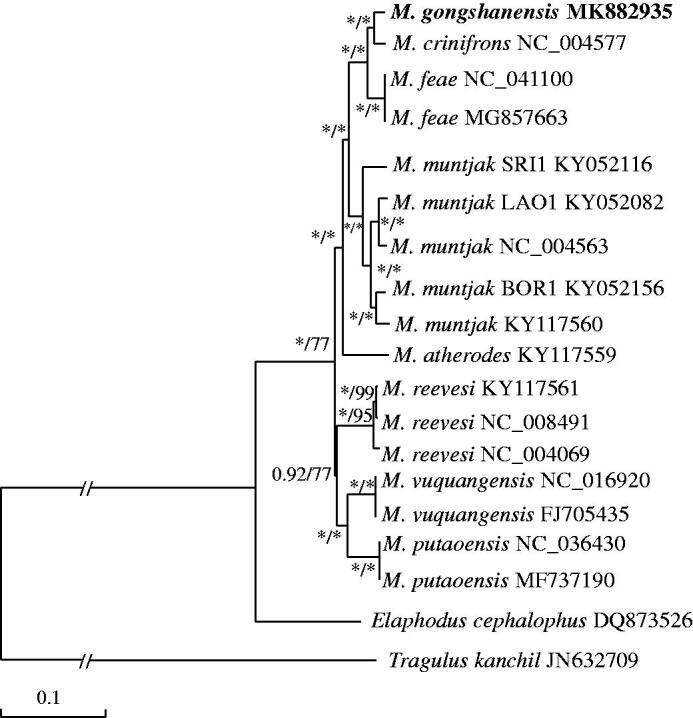
Maximum likelihood (ML) and Bayesian inference (BI) phylogenetic trees for *Muntiacus* based on mitogenome. The mitogenome sequence of the specimen obtained in this work is indicated in bold. Numbers on branches indicate bootstrap supports in Bayesian inference analyses for the node and for ML followed by posterior probability. * indicates values are 1.00 (BI) and 100 (ML).
